# Copulatory Mechanics Reveals a Self‐Bracing Mechanism via a Femoral Apophysis in Funnel Weavers (Araneae, Agelenidae)

**DOI:** 10.1002/ece3.71032

**Published:** 2025-02-23

**Authors:** Alireza Zamani, Rahşen S. Kaya, Kari Kaunisto, Peter Michalik

**Affiliations:** ^1^ Zoological Museum, Biodiversity Unit University of Turku Turku Finland; ^2^ Department of Biology, Faculty of Arts and Science Bursa Uludağ University Bursa Türkiye; ^3^ Zoological Institute and Museum University of Greifswald Greifswald Germany

**Keywords:** Agelenidae, *Anatextrix monstrabilis*, cryofixation, genitalia, micro‐CT, sexual selection, sperm transfer

## Abstract

Spiders utilize an indirect method of sperm transfer via specialized male palpal structures. In entelegyne spiders, these structures exhibit a remarkable complexity, comprising various sclerites that interlock with the female genitalia to provide stability and facilitate sperm transfer. Among the four primary coupling mechanisms recognized in entelegyne spiders, one, termed self‐bracing, involves interactions between structures stabilizing the expanded copulatory organ during mating. Such interactions can involve elements that are not part of the copulatory organ. The retrolateral tibial apophysis (RTA), a characteristic of the largest group of spiders (RTA clade), is the most prominent structure for this purpose. However, recent research has demonstrated that in spiders that have lost the RTA, other parts of the palp, specifically femoral apophyses, can be involved in self‐bracing. The presence of a femoral palpal fapophysis is uncommon in spiders, and only a few taxa possess apophyses on multiple palpal articles, i.e., tibia and femur, the interaction and evolution of which remain to be elucidated. This study investigated the function and interaction of apophyses on different palpal structures for the first time using the funnel weaver *Anatextrix monstrabilis* (Agelenidae). We specifically examined the hypothesis that the various prominent femoral apophyses are involved in self‐bracing despite the presence of an RTA. Micro‐computed tomography data of a cryofixed mating pair revealed that at least one of these apophyses functions in self‐bracing by fitting into the groove of the embolic base, representing the second documented case of this unique self‐bracing mechanism in entelegyne spiders. Furthermore, scanning electron microscopy revealed previously undocumented features in the female genitalia of *Anatextrix*, including an epigynal fovea, an anterior hood, and well‐developed epigynal lateral margins, which potentially play a role in interlocking with male palpal sclerites during copulation. In contrast to ghost spiders (Anyphaenidae), the only other known group of entelegyne spiders exhibiting self‐bracing with femoral apophyses, *Anatextrix* species demonstrate notable differences with regard to the size and shape of these apophyses. Thus, our study indicates that male palpal femoral structures, which do not contact female genitalia during genital coupling, can be subject to strong selection pressures similar to somatic structures that function beyond basic sperm transfer.

## Graphical abstract

In this study, we tested the hypothesis that femoral apophyses function in self‐bracing in *Anatextrix monstrabilis*, one of the two species of a recently discovered lineage of agelenid spiders characterized by a strongly modified male palpal femur. Micro‐computed tomography data of a cryofixed mating pair revealed that at least one of these apophyses functions in self‐bracing by fitting into the groove of the embolic base. Our study represents the second documented case of this unique self‐bracing mechanism in entelegyne spiders.

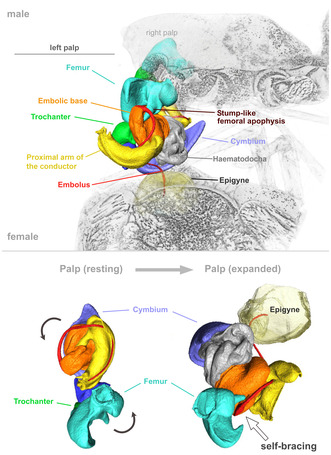

## 1 Introduction

In the animal kingdom, copulatory organs have evolved multiple times as effective mechanisms for direct sperm transfer to females (Hosken and Stockley 2004). These structures serve not only this fundamental purpose but have also developed a diverse array of forms and functions due to intense selective pressures exerted by sexual selection (Leonard and Córdoba‐Aguilar 2010). In this context, it is of paramount importance to distinguish between theories of genital evolution that either postulate the development of structures or physiological features that enable females to discern male characteristics (cryptic female choice) or hypothesize the evolution of anatomical or physiological barriers that impede males from accessing female eggs (sexual conflict) (Brennan 2016). However, understanding the evolutionary dynamics of genital organs, which can be particularly intricate in arthropods (Simmons 2014), largely depends on comprehensive knowledge of the complex mechanical interactions during mating beyond linear estimates of genital size. For example, utilizing micro‐computed tomography (micro‐CT) on in‐copula fixed pairs, Wojcieszek et al. (2012) demonstrated a strong correlation between the diverse male genitalic structures and the complex female reproductive organs, providing essential anatomical evidence to elucidate the significant impact of genital shape variations on intraspecific morphological compatibility in millipedes.

Spiders possess a unique sperm transfer system, with male copulatory organs (copulatory bulbs) situated on the tarsus (cymbium) of the palps, lacking a direct connection to the testes (Eberhard and Huber 2010). Consequently, males must fill both sperm reservoirs (spermophor) in their copulatory bulbs with sperm prior to mating (see Foelix 2011). During copulation, sperm is transferred to females through the insertion of the intromittent sclerite (embolus) into the female copulatory opening (palpal insertion). The few available studies have demonstrated that, as observed in other arthropod taxa such as insects, genitalic interactions in spiders can exhibit considerable complexity depending on the degree of complexity of male and female genitalia and adjacent structures (e.g., Eberhard and Huber 2010; Poy et al. 2023a, 2023b).

The vast majority of spiders possess an entelegyne‐type reproductive system, wherein females exhibit an externally sclerotized genital area (epigyne) and relatively complex internal structures with distinct ducts for insemination and fertilization (see Foelix 2011). Males possess correspondingly complex copulatory bulbs with various sclerites interconnected by membranous areas (hematodochae). During copulation, hydraulic expansion of the hematodochae facilitates a sequential interlocking of the male copulatory structures into corresponding structures of the epigyne (Eberhard and Huber 2010). The conductor and median apophysis are two common bulbal sclerites observed in entelegyne spiders; the former typically guides or accommodates a portion of the embolus during copulation.

The majority of entelegyne groups possess additional structures on the bulb or palpal segments that provide stability. The most diverse group of entelegyne spiders, the RTA clade, is characterized by the presence of a retrolateral tibial apophysis (RTA) on the male palp, which, during copulation, facilitates precise positioning of the male copulatory organ in relation to the copulatory openings of the female (Huber 1995a). Currently, four types of mechanisms are recognized in the coupling of entelegyne spiders (Poy et al. 2023a): (1) primary locking, which refers to the initial contact between the male copulatory organ and the epigyne, occurring before hematodochal expansion and facilitated primarily by the RTA or, if absent, by other sclerites such as the median apophysis; (2) secondary locking, during which hematodochal expansion and further interlocking between copulatory structures occur; (3) functional conduction, where the embolus is guided through one of the female copulatory openings; and (4) self‐bracing, involving mechanical interactions between male genital structures to further stabilize the inserted palp. In entelegyne spiders, only a few exceptions are known where self‐bracing is achieved using tibial apophyses, either with the retrolateral tibial apophysis (RTA) itself or the ventral tibial apophysis (see summary in Poy et al. 2023a). A notable feature for entelegyne spiders was recently discovered: a self‐bracing mechanism utilizing femoral apophyses in species of ghost spiders (Anyphaenidae) that have lost the RTA. This finding was significant as it demonstrated that femoral elements can also play a substantial role in the genital coupling of entelegyne spiders by stabilizing the male copulatory organ during copulation without having contact with the female genitalia, as usually facilitated by the RTA during primary locking. In light of these new findings, it is of particular interest to elucidate the function of such femoral structures present in species that have not lost the RTA, providing a contrasting scenario for comparison.

Here, we examined a species of the funnel weaver genus *Anatextrix* Kaya et al. 2023, which was recently described from southern Anatolia (Kaya et al. 2023a, 2023b). *Anatextrix* is characterized by a significantly modified male palpal femur, bent at either the proximal or middle part, bearing two apophyses and at least one bulge. The tibia, in addition to an RTA, possesses a small prolateral apophysis and a ventral keel, while the patella bears a ventral apophysis. This combination of characters appears to be unique among spiders (Kaya et al. 2023b), rendering *Anatextrix* an ideal study system to investigate the function of non‐copulatory structures, including femoral apophyses, in detail. Given that the RTA is present in both currently known species of *Anatextrix* and is presumably involved in primary locking, we hypothesized that the femoral apophyses are only involved in self‐bracing, similar to what has been previously documented by Poy et al. (2023a). To test this hypothesis, we analyzed the copulatory interactions of *A. monstrabilis* Kaya et al. 2023 utilizing micro‐CT of a cryofixed mating pair and by employing scanning electron microscopy (SEM) images of the epigyne and male palpal structures.

## 2 Material and Methods

### 2.1 Collection and Rearing of Specimens

*Anatextrix monstrabilis* inhabits montane forests in the Eastern Taurus Mountains in Türkiye. For this study, six adult specimens, consisting of three males and three females, were collected in October 2023 from a pine forest in Pozantı Akça Tekir Plateau, located in the Pozantı District of Adana Province. The specimens were collected from their funnel webs, which were constructed under rocks and in soil crevices.

Each individual was housed separately in a small plastic enclosure (5 × 5 × 5.5 cm) at room temperature (25°C) for 2 weeks, during which time they constructed small sheet webs. The webs were lightly misted with water every 2 days, and the spiders were fed twice per week with fruit flies ( *Drosophila melanogaster* Meigen, 1830).

### 2.2 Cryofixation of Mating Couples

For mating, males were removed from their enclosures and introduced to the webs of the females. Pairs engaged in copulation were cryofixed by pouring liquid nitrogen (−195.8°C) directly over them during a palpal insertion, after which they were immediately transferred to cold 80% ethanol (−18°C).

### 2.3 Visualization of the Samples

Video footage of mating couples was taken from above, using a Sony α9 Mark II camera, equipped with a Canon 100 mm f/2.8 L lens, mounted via a Sigma MC‐11 adapter. The successfully cryofixed couple was investigated with X‐ray microscopy. The dehydrated specimens were stained in a 1% iodine solution for 12 h and then washed in 100% ethanol. The scans were performed with an Xradia MicroXCT‐200 X‐ray imaging system (Carl Zeiss Microscopy GmbH) using the ×10 lens. Tomographic projections were reconstructed using the XMReconstructor software (Carl Zeiss Microscopy GmbH). Segmentation and virtual reconstructions of the image stacks were performed using Amira 6.4 (Thermo Fisher Scientific Inc.).

For SEM photography, the samples were cleaned and dried for 15 min. Each sample was then attached to an aluminum stub using a sticky carbon disc (Leit Adhesive Carbon Tabs, 12 mm) as a conductive adhesive. Subsequently, the samples were sputter‐coated with a thin layer of gold using a sputter coater. The prepared samples were placed in the chamber of a JSM‐6490LV SEM, where the imaging parameters—including accelerating voltage, working distance, and magnification—were adjusted to optimize image quality. During image acquisition, appropriate scan modes were employed. Initial focusing was performed using a fast scan mode, followed by slower, higher‐resolution scans to capture the final images.

A digital image of the cryofixed pair was obtained using an Olympus Camedia E‐520 camera attached to an Olympus SZX16 stereomicroscope.

## 3 Results

### 3.1 Mating Process

Only one cryofixation attempt was successful. In one pair, the individuals became separated during the cryofixation process, while in another, the male killed the female. In the successfully cryofixed pair, although the embolus remained inside the female genitalia, the rest of the palp was not in contact with the female genitalia, seemingly due to a slight detachment that occurred during the fixation process. Specimens from the second pair were used for scanning electron microscopy.

**FIGURE 1:**
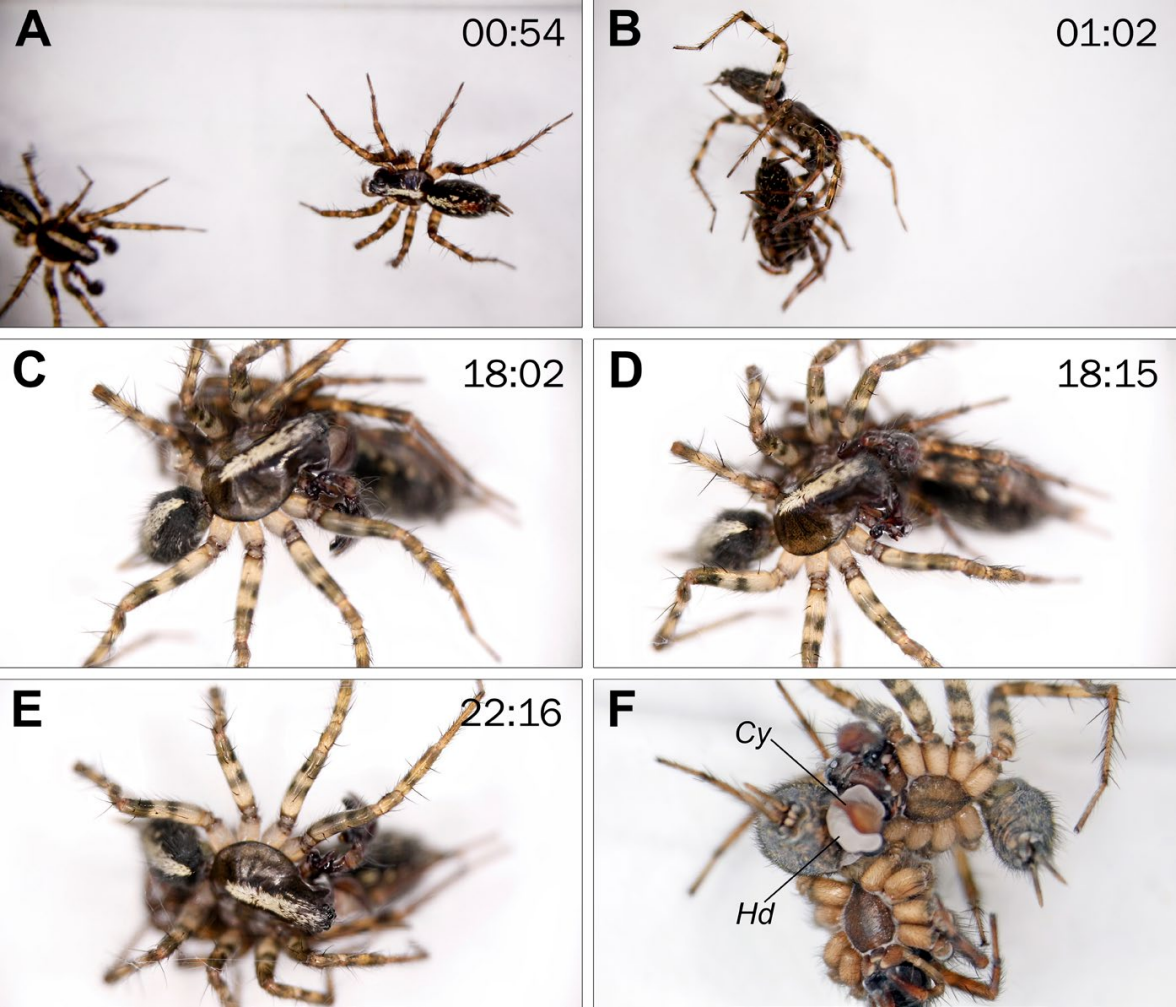
Mating sequence of *Anatextrix monstrabilis*, with the time elapsed since the male's introduction into the female's enclosure indicated. (A) Male (left) approaching the female. (B) Male grasping the patella of the female's left fourth leg with his chelicerae. (C) First palpal insertion. (D) Palpal cleaning. (E) Second palpal insertion. (F) Cryofixed pair. Abbreviations: Cy, cymbium; Hd, hematodocha.

In the successful attempt, the male approached the female from the front almost immediately after being introduced to her enclosure (Figure 1A). Upon sensing his approach, the female swiftly attempted to attack, resulting in the pair briefly tangling. Shortly afterward, the male was observed holding the patella of her left fourth leg with his chelicerae, while the female relaxed and vibrated her abdomen for a few seconds (Figure 1B). The male then shifted his position, moved over the female, and carried her around for a few minutes by holding onto her left first leg. During this time, the position of the female alternated between lying on her side and returning to the original position.

The male then began palpating the nearly motionless female, followed by cleaning his palps with his chelicerae, and then more intense palpating. He also gently pushed the female using his body and palp to raise her slightly into a position suitable for copulation. Shortly afterwards, a hematodochal expansion and a brief insertion occurred in the left copulatory opening (Figure 1C), but the male quickly resumed cleaning his palps for about 2 min (Figure 1D). After this, the male changed the position of the female again and performed a brief insertion using his right palp (Figure 1E), which lasted only a few seconds and was followed by more palpal cleaning. The subsequent insertion was done also using the right palp, during which the cryofixation was performed. In the fixed position, the male and female were positioned nearly side by side, facing in almost opposite directions (Figure 1F).

### 3.2 Genital Morphology

**FIGURE 2:**
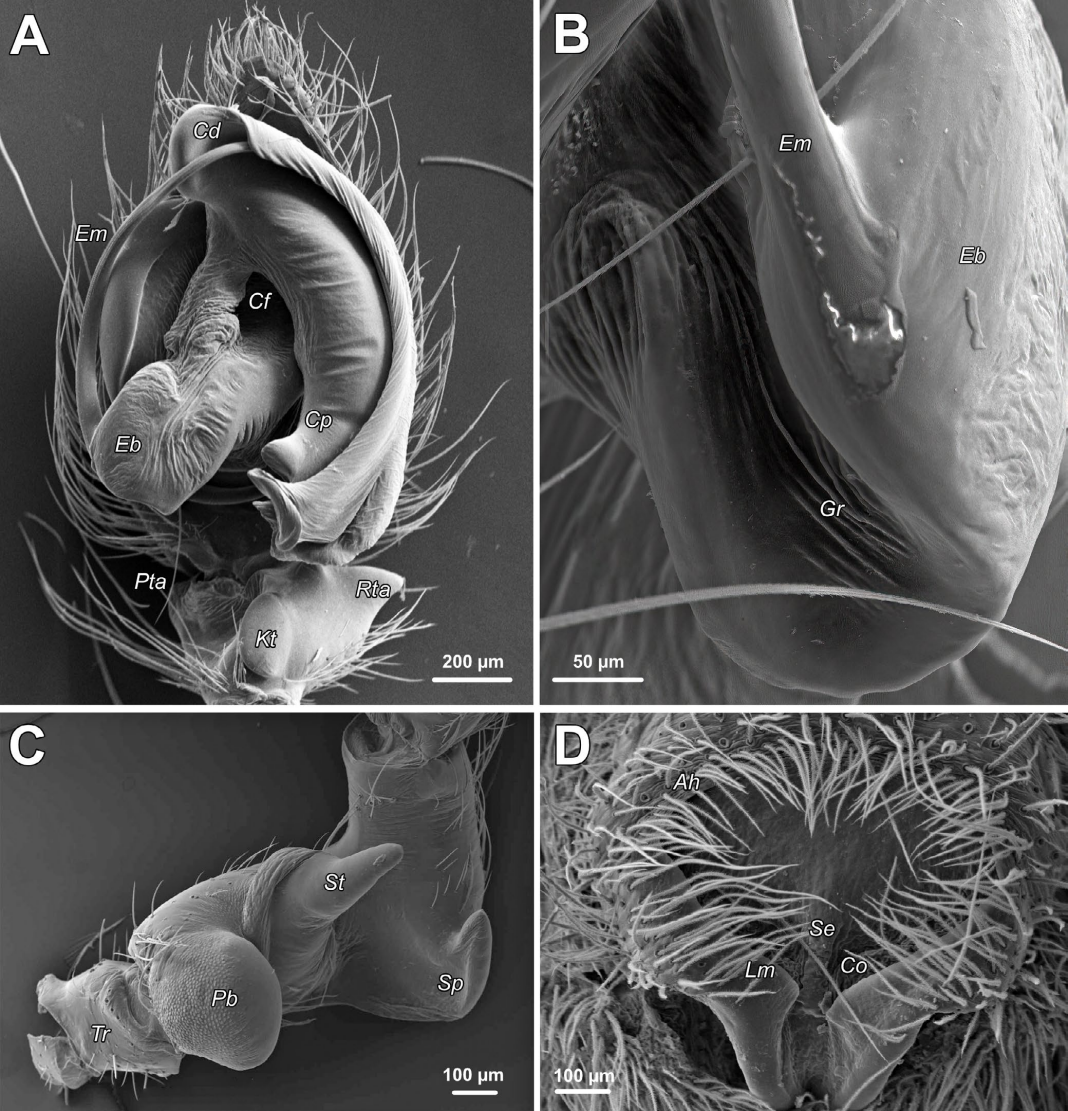
SEM images of the copulatory organs of *Anatextrix monstrabilis*. (A–C) Left male palp. (D) Epigyne. (A) Tibia, cymbium, and bulb, ventral view. (B) Groove of the embolic base, prolateral view. (C) Femur and trochanter, retrolateral view. (D) Intact, posteroventral view. Abbreviations: Ah, anterior hood; Cd, distal arm of the conductor; Cf, furrow of the conductor; Co, copulatory opening; Cp, proximal arm of the conductor; Eb, embolic base; Em, embolus; Gr, groove of the embolic base; Kt, ventral keel; Lm, lateral margin; Pb, proximal bulge; Pta, prolateral tibial apophysis; Rta, retrolateral tibial apophysis; Se, septum; St, stump‐like femoral apophysis; Sp, spine‐like femoral apophysis; Tr, trochanter.

The male palpal segments of *Anatextrix monstrabilis* are highly modified. The femur is sharply bent at a right angle in the mesal region and has a proximal retrolateral bulge (*Pb*) as well as two apophyses: a stump‐like femoral apophysis (*St*) located on the retroventral surface of the “horizontal” portion of the femur, and a spine‐like femoral apophysis (*Sp*) on the retrolateral surface at the base of the “vertical” portion (Figure 2C). The patella has a ventral apophysis (Kaya et al. 2023b: Figure 2A). The tibia has a small, laterally directed conical retrolateral apophysis (*Rta*), a ventral keel (*Kt*), and a minute prolateral apophysis (*Pta*). The conductor is large, consisting of a proximal arm (*Cp*) and a distal arm (*Cd*), with the former bearing three terminal outgrowths (Figure 2A). The embolic base (*Eb*) is relatively broad and has a distinct groove (*Gr*; Figure 2B). The embolus originates at the 8:30 position and, from the 12:00 position onward, fits into a cavity formed by a fold within the conductor (Figure 2A).

The epigynal plate is relatively wide and has a deep fovea. The epigyne has a thin septum (*Se*) posteriorly, with copulatory openings (*Co*) positioned on either side. It also has slanting lateral margins (*Lm*) that converge posteriorly, while anteriorly forming a broad hood (*Ah*) with a curved margin (Figure 2D). For details on the internal structures of the female genitalia, see Kaya et al. (2023b).

### 3.3 Copulatory Mechanics

**FIGURE 3:**
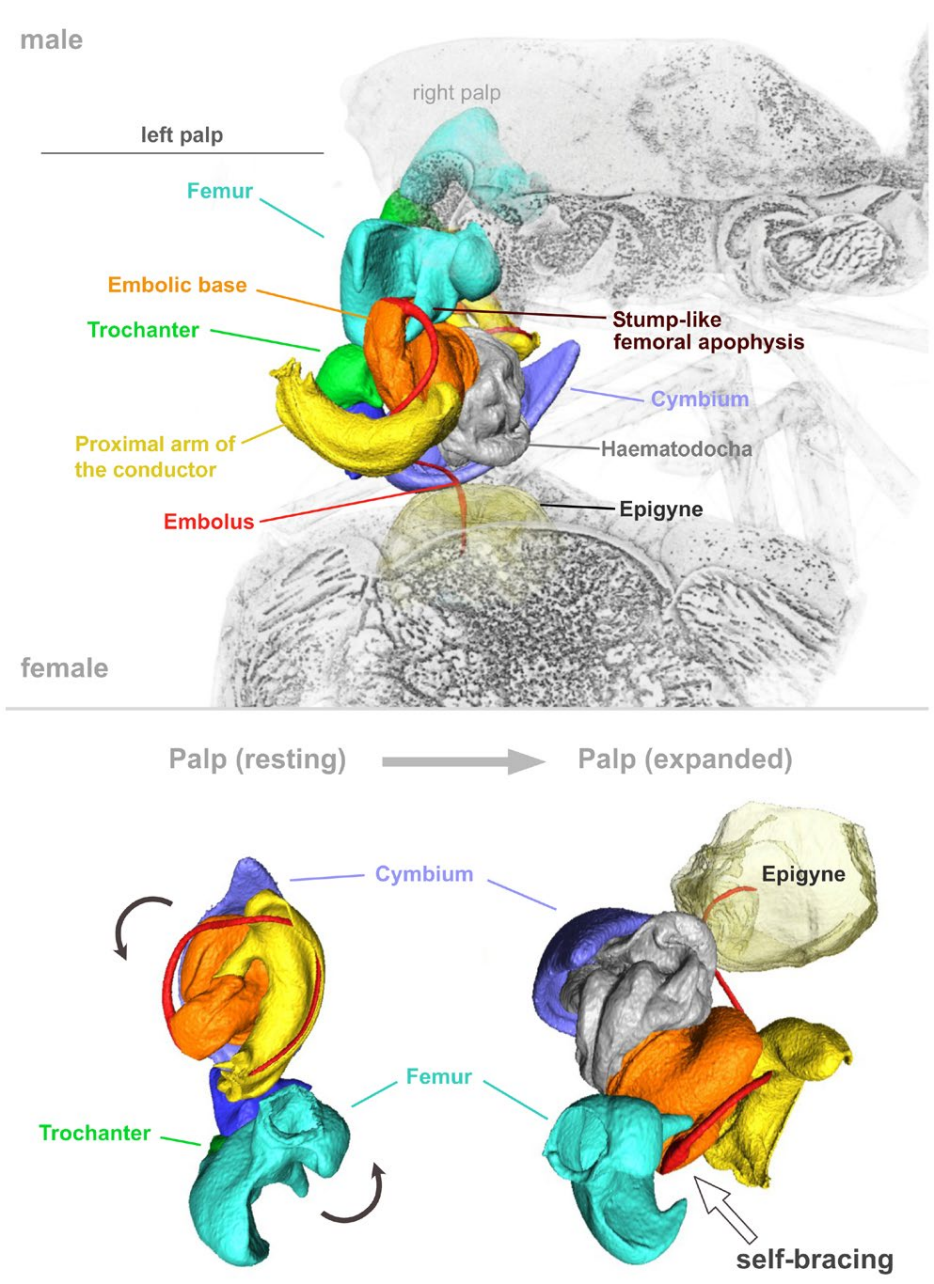
Virtual reconstruction of the copulatory mechanism of *Anatextrix monstrabilis*. The expanded left palp is stabilized by self‐bracing, which is achieved by locking the stump‐like femoral apophysis into the groove of the embolic base.

The genital interactions during primary locking are unclear, as the specimens were cryofixed after the hematodochae had already expanded. However, the small RTA does not seem to play a significant role during primary locking based on its small size. Since the bulb was slightly detached from the epigyne during the preparation process, the interactions involved in secondary locking also remain unknown, though they may be absent, as there are no apophyses on the bulb. Alternatively, this could be attributed to the fact that palpal insertion lasts only a few seconds, preventing the formation of a strong anchoring mechanism. In functional conduction, the embolus is guided through the furrow of the conductor (*Cf*) into the copulatory opening. Self‐bracing was achieved by the stump‐like femoral apophysis sliding into the groove of the embolic base (Figure 3).

## 4 Discussion

Research on self‐bracing mechanisms in Entelegynae is limited. In the most diverse group of spiders, the RTA clade, self‐bracing can occur between tibial structures and the bulb, while in the superfamily Araneoidea, it involves the paracymbium or, less frequently, the cymbial hood (for a review, see Poy et al. 2023a). The sole previously documented instance of self‐bracing via femoral apophyses in Entelegynae was reported from the anyphaenid genus *Josa* Keyserling, 1891, which lacks the RTA. Outside Entelegynae, similar interactions involving a femoral apophysis have been previously documented in a few species of Pholcidae (Huber 1998).

This study demonstrates a self‐bracing function in a species with an RTA for the first time, utilizing the prominent stump‐like femoral apophysis by fitting into the groove of the embolic base, thereby providing stability to the male copulatory organ during copulation. Furthermore, the spine‐like femoral apophysis did not interlock with any structure, though based on its position, it likely fits into the cavity at the terminal part of the proximal arm of the conductor, contributing further to the stability of the expanded copulatory organ. Although documentation of the interactions during primary locking was not feasible, it can be hypothesized that the retrolateral side of the cymbium would align with the posterior part of the epigyne, with the retrolateral portion of the conductor and the RTA fitting into its lateral margins to contribute to stabilizing genital coupling. The involvement of the RTA in stabilizing through self‐bracing has also been described for the agelenid genus *Agelenopsis* Giebel, 1869, where this apophysis either supports the median apophysis of the bulb or limits cymbial flexure (Gering 1953). Additionally, while the detachment of the expanded palp could be attributed to the preparation process, it might also be due to a weaker secondary locking resulting from the absence of prominent bulbal and tibial apophyses compared to other RTA groups (e.g., Huber 1995a, b; Poy et al. 2019; Poy et al. 2023b). Further in‐depth studies are necessary to elucidate the detailed interactions of the male and female genitalia in *Anatextrix* and related taxa.

Poy et al. (2023a) demonstrated that in contrast to genital structures, the femoral apophysis exhibits relative uniformity in *Josa* males. Consequently, they postulate that the femoral apophysis is either subject to a weak directional selective regime compared to structures that interact with the female during genital coupling or, alternatively, under a strong stabilizing selective regime (Poy et al. 2023a). Regarding the genus *Anatextrix*, the situation is less definitive, as the femoral apophyses also vary in shape and size. Although *A. spectabilis* Kaya et al. 2023 possesses a similar stump‐like femoral apophysis, which likely serves a comparable function during self‐bracing, the femoral bend is situated proximally and is less pronounced than in *A. monstrabilis*. Furthermore, the spine‐like apophysis is smaller in *A. spectabilis* and presumably does not contribute to self‐bracing, though its potential role in *A. monstrabilis* remains undetermined. Considering the different elements of the *Anatextrix* male palp, it is remarkable that the RTA is much less pronounced compared to the femoral modification (Kaya et al. 2023a, 2023b). Thus, it can be hypothesized that *Anatextrix* represents an intermediate stage with a strong stabilizing selective regime on femoral structures and relaxed selection on tibial apophyses due to changes in the copulatory mechanics (see discussion in Poy et al. 2023a). In fact, males of *Anatextrix* are very remarkable for possessing two femoral apophyses and at least one proximal bulge. The strange femoral bend appears to facilitate the self‐bracing function by positioning the apophyses closer to the bulb. Additionally, comparable to *Josa* and a few other genera with femoral modifications, such as *Maimuna* Lehtinen, 1967 of the tribe Textricini, to which *Anatextrix* also belongs (Levy 1996; Poy et al. 2023a; Zamani et al. 2024), males of *Anatextrix* have a short palpal tibia and patella, which further facilitate the interaction of femoral structures with the bulb.

In summary, our study shows that male palpal femoral structures, which are not in contact with female genitalia during genital coupling, can be under strong selection similar to somatic structures that function beyond basic sperm transfer. Examples of such structures are manifold in the animal kingdom, including claspers (as in water striders, see Ronkainen et al. 2005), sperm removal devices (as in many dragonflies, see Córdoba‐Aguilar et al. 2003) and titillators (as in bush crickets, see Wulff et al. 2015). In the case of *Anatextrix*, the femoral structures facilitate a strong stabilization of the expanding palp during sperm transfer and thus contribute to the mechanical fitness of the male genitalia, which are likely under stronger selection compared to the rather inconspicuous female genital structures, as it was also hypothesized for other animal taxa (e.g., Genevcius and Schwertner 2017). To elucidate the distinctions between the evolutionary mechanisms, subsequent investigations should focus on elucidating the factors that favor a robust and stable coupling and determining whether such coupling is cooperative or antagonistic in nature.

## Author Contributions

**Alireza Zamani:** conceptualization (equal), funding acquisition (lead), methodology (equal), visualization (equal), writing – original draft (lead), writing – review and editing (equal). **Rahşen S. Kaya:** methodology (equal). **Kari Kaunisto:** methodology (equal), visualization (equal). **Peter Michalik:** conceptualization (equal), methodology (equal), visualization (equal), writing – original draft (supporting), writing – review and editing (equal).

## Conflicts of Interest

The authors declare no conflicts of interest.

## Data Availability

All data presented are included in the manuscript.
